# Toward clinical and molecular understanding of pathogenic variants in the *ZBTB18* gene

**DOI:** 10.1002/mgg3.387

**Published:** 2018-03-24

**Authors:** Vyne van der Schoot, Sonja de Munnik, Hanka Venselaar, Mariet Elting, Grazia M. S. Mancini, Conny M. A. Ravenswaaij‐Arts, Britt‐Marie Anderlid, Han G. Brunner, Servi J. C. Stevens

**Affiliations:** ^1^ Department of Clinical Genetics Maastricht University Medical Centre Maastricht the Netherlands; ^2^ Department of Human Genetics Radboud University Medical Centre Nijmegen Nijmegen the Netherlands; ^3^ Centre for Molecular and Biomolecular Informatics (CMBI) Radboud University Medical Centre Nijmegen Nijmegen the Netherlands; ^4^ Department of Clinical Genetics Vrije Universiteit Amsterdam the Netherlands; ^5^ Department of Clinical Genetics Erasmus MC University Medical Centre Rotterdam the Netherlands; ^6^ University of Groningen University Medical Centre Groningen Department of Genetics Groningen the Netherlands; ^7^ Department of Clinical Genetics Karolinska Universitetssjukhuset Solna Sweden

**Keywords:** C2H2zinc finger (ZNF) domain, corpus callosum anomalies, homology modeling, intellectual disability, *ZBTB18*

## Abstract

**Background:**

Patients with pathogenic variants in *ZBTB18* present with Intellectual Disability (ID) with frequent co‐occurrence of corpus callosum (CC) anomalies, hypotonia, microcephaly, growth problems and variable facial dysmorphologies. These features illustrate a key role for *ZBTB18* in brain development.

**Methods:**

Patients with a pathogenic variant in *ZBTB18* were detected by diagnostic whole exome sequencing (WES) performed in our center. We reviewed the literature and used GeneMatcher to include other cases. YASARA and WHAT IF were used to provide insight into the structural effect of missense variants located in the C2H2 zinc finger domains of the ZBTB18 protein.

**Results:**

We give a complete overview of pathogenic variants in *ZBTB18* detected to date, showing inconsistent presence of clinical features, including CC anomalies. We present four new cases with a de novo pathogenic variant in the *ZBTB18* gene, including the fourth case in which a de novo p.Arg464His variant was found.

**Conclusion:**

Homology modeling of protein structure points to a variable degree of impaired DNA binding caused by missense variants in these domains probably leading to Loss of Function (LoF). Putative partial LoF may present with a less distinctive phenotype than complete LoF, as seen in truncating variants, which presents with an extensive variability in the phenotypic spectrum. Our data do not support a clear genotype to phenotype correlation.

## 1 INTRODUCTION

Patients with a 1q43q44 microdeletion (OMIM# 612337) present with variable intellectual disability (ID), possible agenesis of the corpus callosum (ACC) and variable microcephaly. The *ZBTB18* gene (*ZNF238*; OMIM# 608433) has previously been identified as contributing factor for the 1q43q44 deletion syndrome phenotype. Some of the features seen, such as CC anomalies have been linked to haploinsufficiency of the *ZBTB18* gene (Ballif et al., 2012). Seizures seen in some cases with a 1q43q44 deletion are explained by loss of the *HNRNPU* (OMIM# 602869) gene (Depienne et al., 2017; Hamdan et al., 2014; Hemming et al., 2016; de Kovel et al., 2016). Dysmorphic facial features (e.g., hypertelorism, strabismus, prominent nasal tip, bulbous nose, abnormal philtrum or lips, micro‐ or retrognathia, abnormal ears) and other clinical features, such as growth problems are inconsistently described in these patients and have not been linked to a specific gene in the 1q43q44 region yet.

Patients with a pathogenic variant in the *ZBTB18* gene show phenotypic overlap with 1q43q44 microdeletion syndrome patients. Multiple case reports and patient series have been published to delineate the clinical phenotype of the *ZBTB18*‐ related disorder (Cohen et al., 2017; Depienne et al., 2017; de Munnik et al., 2014). In approximately half of the described cases, CC anomalies, hypotonia, microcephaly, growth problems and variable facial dysmorphologies were reported as well (Cohen et al., 2017). Presumably, pathogenic variants in *ZBTB18* cause variable CC anomalies with a reduced penetrance (Depienne et al., 2017).

The aim of this study was to further delineate phenotypic spectrum of patients with de novo *ZBTB18* variants, to establish a putative genotype‐phenotype correlation and to characterize the putative molecular effect of *ZBTB18* missense variants on protein function, using homology‐based‐modeling. We present four new cases with a de novo pathogenic variant in the *ZBTB18* gene and review 21 previously described cases.

## 2 MATERIALS AND METHODS

### 2.1 Ethical compliance

Written informed consent of the patients' parents was obtained before inclusion in the exome‐ sequencing study/diagnostic exome sequencing. This study was approved by the local institutes under the realm of routine diagnostic genetic testing. Patients' parents were counseled by a clinical geneticist and gave written informed consent for the diagnostic procedure. As a result, these patients or their families were not subjected to additional investigations for the purposes of research. No formal ethical board review was required for this retrospective research/patient file research type of study, since data are already available from routine diagnostic testing.

### 2.2 Exome sequencing

Routine diagnostic whole exome sequencing (WES) in patients with ID from the University Medical centers in Maastricht and Nijmegen identified three patients with a de novo variant in the *ZBTB18* gene. Written informed consent was obtained from patients' parents for exome sequencing and written permission was obtained for inclusion of clinical photographs (where applicable).

The WES procedure was performed, using the previously described trio parent‐offspring approach (Veltman & Brunner, 2012). DNA was isolated from blood according to standard procedures. Exome capturing was performed, using the Agilent SureSelect v4 kit (Agilent, Santa Clara, CA, USA) and exome libraries sequencing was performed, using an Illumina HiSeq instrument (Illumina, San Diego, CA, USA) with 101 bp paired‐end reads at a median coverage of 75×. BWA version 0.5.9‐r16 was used to align sequence reads to the hg19 reference after which the GATK unified genotyper, version 3.2‐2 was used to call variants, combined with annotation, using a custom diagnostic annotation pipeline. Calling of de novo variants in index patients was performed according to de Ligt et al. (2012). To validate these de novo variants, standard Sanger sequencing was performed on patients and parental DNA.

Using genematcher (https://genematcher.org; Sobreira, Schiettecatte, Valle, & Hamosh, 2015) we identified a fifth patient with a de novo *ZBTB18* variant. Literature was reviewed to include patients previously described.

### 2.3 Molecular modeling of *ZBTB18* missense variants

To obtain insight into the putative effect of the missense variants on the molecular structure of the ZBTB18 protein, a homology model was created. We used the extensively validated YASARA (Krieger, Koraimann, & Vriend, 2002) and WHAT IF twinset homology scripts (Vriend, 1990). Since no crystal structure of ZBTB18 is available, PDB file 5K17 was used to create a homology model for amino acid residues 430–500, which are ~40% identical to the homologue. The effect of the missense variants on ZBTB18 protein structure was analyzed using standard biochemical amino acid characteristics (i.e., chemical structure and size, polarity of the side chain, charge, and isoelectric point) and knowledge from the Uniprot database (The UniProt Consortium, 2017). Variants were identified by comparison with the NCBI reference protein sequence NP_991331.1 encoded by transcript NM_205768.2 (http://www.ncbi.nlm.nih.gov/gene) and described according to HGVS guidelines.

## 3 RESULTS

### 3.1 Clinical data and variants in *ZBTB18*

We present four new cases with a de novo *ZBTB18* variant. Patient 1, a 15‐year‐old boy, presented with developmental delay, hypotonia, and seizures. His head circumference (OFC) was 52 cm (−2.25SD). His height was 160 cm (−2SD). MRI did not reveal corpus callosum abnormalities, nor other structural brain anomalies. He had a prominent and bulbous nose and showed strabismus. He carried a de novo missense variant Chr1(GRCh37):g.244218415T>C, NM_205768.2(ZBTB18):c.1339T>C (p.(Tyr447His)). This variant is predicted to be probably damaging (Polyphen score 0.969) and deleterious (SIFT score 0) and affects a conserved amino acid residue (conserved up to fish) located in a C2H2 zinc finger (ZNF) domain of the ZBTB18 protein. This variant has not been described in individuals from the ExAC database (exac.broadinstitute.org).

**Figure 1:**
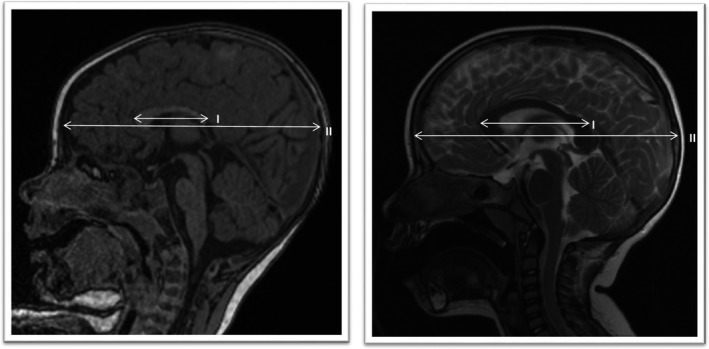
Midsagittal MRI images. Left: Patient 1. One‐year‐old boy with corpus callosum dysgenesis. T1‐FLAIR. Width I:II = 1:3.6. Right: Normal control. Two‐year‐old boy. T2‐FLAIR. Width I:II = 1:2.2. Case courtesy of *Dr Bruno Di Muzio*, Radiopaedia.org

Patient 2, a 1‐year‐old boy, presented with microcephaly (OFC 43.5 cm; −2.8 *SD*) and attacks of abnormal arm extension. His motor and speech development were delayed. He showed truncal hypotonia on physical examination. His length was 84 cm, he weighed 10 kg. EEG results were normal. MRI showed a short and hypoplastic corpus callosum of which the splenium was affected more than the rostrum (Figure 1). He had an upward slant, a small and somewhat sloping forehead, depressed nasal bridge, small and upturned nose tip and nostrils, elongated philtrum and a thin upper lip. A de novo nonsense variant was found: Chr1(GRCh37):g.244217655G>A, NM_205768.2(ZBTB18): c.579G>A (p.(Trp193*)) that leads to a premature stop codon.

Patient 3, a 13‐year‐old boy, presented with mild to moderate speech and developmental delay and attention deficit disorder (ADD). He did not have hypotonia. His OFC was 52.5 cm (−1.25 *SD*). He was 156 cm tall (−0.75 *SD*). No structural brain anomalies were seen on MRI. He had retrognathia, mild hypertelorism, and a slightly elongated philtrum and thin upper lip. His hands were broad and short. Mild syndactyly of the second and third toe with a sandal gap were seen in both feet. WES analyses showed a de novo frameshift variant Chr1(GRCh37):g.244217335del, NM_205768.2(ZBTB18):c.259del(p.(Leu87Cysfs*21)), that leads to a premature termination codon located more than 400 codons upstream of the canonical termination codon.

Patient 4, a 4‐year‐old boy, presented with severe speech delay, motor delay, and hypotonia. MRI showed agenesis of the splenium of the corpus callosum. At 3 years of age, an OFC of 49 cm was measured (−1 *SD*). His height was 98 cm (0 *SD*). He had hypertelorism, a prominent nasal tip, and a bulbous nose, a small mouth and retro‐ and micrognathia. His fingers showed broad tips. He carried a missense variant in *ZBTB18* (Chr1(GRCh37):g.244218467G>A, NM_205768.2(ZBTB18):c.1391G>A(p.Arg464His)). This heterozygous de novo missense variant is predicted to be deleterious (SIFT score 0; Polyphen score 0.991) and affects a highly conserved amino acid residue located in the ZNF domain of the ZBTB18 protein (conserved up to *Tetraodon*). This variant has not been found in individuals from the ExAC database.

**Figure 2:**
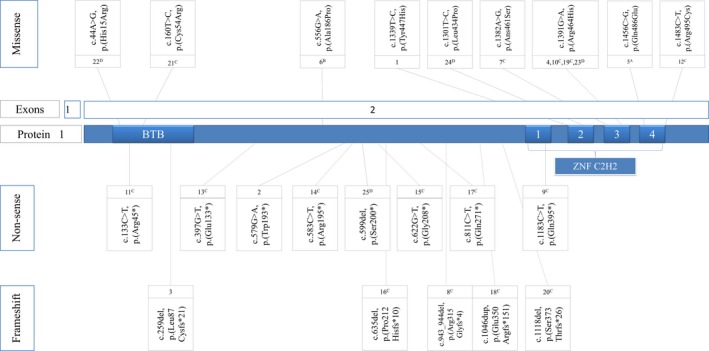
Pathogenic variants in *ZBTB18* (ref. transcript NM_205768.2) with Zn fingers (1–4) in exon 2. Numbers corresponding with Table 1. (A) Rauch et al., 2012; (B) Lopes et al., 2016; (C) Cohen et al., 2017; (D) Depienne et al., 2017

We reviewed four patient cohorts containing one or more patients with pathogenic variants in *ZBTB18* (Cohen et al., 2017; Depienne et al., 2017; Lopes et al., 2016; Rauch et al., 2012) and included one case report (de Munnik et al., 2014). So far, a total of 25 patients with a pathogenic *ZBTB18* variant have been reported in literature and in this study. All patients presented with developmental delay in varying degrees with prominent speech delay. Fifteen patients underwent an MRI scan. Nine of them showed corpus callosum abnormalities. Results of clinical evaluation of congenital anomalies in 13 patients were present: dysmorphic facial features were seen in 10 patients, epilepsy was described in five patients, hypotonia in seven, and dystonia in two. Data about growth, development, neurological, or congenital anomalies was incomplete in 13 cases. Clinical data of cases included in this study and patients from literature are presented in Table 1. Variants in the *ZBTB18* gene are schematically depicted in Figure 2.

**Table 1:** Clinical characterization of patients carrying a pathogenic variant in *ZBTB18* (ref. transcript NM_205768.2) described so far

Patient nr. (corresponding Figure 3)	This study				Cohen et al., 2017																	Depienne et al., 2017				Total^a^
	1	2	3	4	5	6	7	8	9	10	11	12	13	14	15	16	17	18	19	20	21	22	23	24	25	
	p.(Tyr‐447His)	p.(Trp‐193^a^)	p.(Leu‐87Cysfs^a^21)	p.(Arg‐464His)	p.(Gln‐486Glu)	p.(Ala‐186Pro)	p.(Ans‐461Ser)	p.(Arg‐315Glyfs^a^4)	p.(Gln‐395^a^)	p.(Arg‐464His)	p.(Arg‐45^a^)	p.(Arg‐495Cys)	p.(Glu‐133^a^)	p.(Arg‐195^a^)	p.(Gly‐208^a^)	p.(Pro‐212Hisfs^a^10)	p.(Gln‐271^a^)	p.(Glu‐350Argfs^a^15)	p.(Arg‐464His)	p.(Ser‐373Thrfs^a^26)	p.(Cys‐54Arg)	p.(His‐15Arg)	p.(Arg‐464His)	p.(Leu‐434Pro)	p.(Ser‐200^a^)	
Sex	M	M	M	M	F	F	M	M	M	M	M	F	F	F	M	F	M	M	F	M	U	M	F	M	F	
Age at examination (years)	15.0	1.0	13.0	4.0	U	U	7.0	3.0	4.0	34.0	6.0	18.0	2.0	5.0	U	U	U	U	U	15.0	U	14.0	12.0	23.0	12.0
Growth at examination (Hall, Allanson, Gripp, & Slavotinek, 2007)																										**7/17**
Height (cm)	160.0	84.0	156.0	98.0	U	U	136.0	90.5	98.0	178.0	122.0	169.0	79.0	U	U	U	U	U	U	U	U	172.0	142.0	192.0	155.5	Microcephaly
Height (SD)	−1.0	1.0	0.0	−1.5	U	N	2.0	−1.0	−1.0	0.0	1.0	0.0	<−2	N	U	U	U	U	U	N	U	1.0	−1.8	3.0	1.0	
Weight (kg)	47.5	10.0	40.0	15.0	U	U	23.9	12.7	13.2	N	20.6	57.0	43.0	U	U	U	U	U	U	U	U	44.0	29.5	U	54.9	
Weight (SD)	0.5	−1.0	−1.0	−1.3	U	U	0.0	−1.0	0.0	0.0	0.0	1.0	<−2	N	U	U	U	U	U	N	U	−0.3	−1.5	U	2.0	
OFC (cm)	52.0	43.5	52.5	49.0	U	U	50.5	47.5	47.2	58.0	50.0	U	U	U	U	U	U	U	U	U	U	54.5	50.0	54.5	54.0	
OFC (SD)	−**2.0**	−**2.8**	−1.0	−1.8	U	**<**−**2**	−1.0	−1.0	−**2.0**	1.0	−1.0	U	U	**<**−**2**	U	U	U	U	U	N	**<**−**2**	0.0	−**2.3**	−1.0	0.5	
Development																										25/25
Cognitive delay	Yes	Yes	Yes	Yes	Yes	Yes	Yes	Yes	Yes	Yes	Yes	Yes	Yes	Yes	Yes	Yes	Yes	Yes	Yes	Yes	Yes	Yes	Yes	Yes	Yes	Devel. delay
Motor delay	U	Yes	U	U	U	Yes	Yes	Yes	Yes	Yes	No	Yes	Yes	Yes	Yes	Yes	Yes	Yes	Yes	U	U	Yes	Yes	Yes	Yes	
Speech delay	Yes	Yes	Yes	Yes	U	Yes	Yes	Yes	Yes	Yes	Yes	Yes	Yes	Yes	Yes	Yes	Yes	Yes	Yes	Yes	Yes	Yes	Yes	Yes	Yes	
Neurology																										9/15
Corpus callosum abnormalities	No	**Yes**	No	**Yes**	U	No	**Yes**	**Yes**	**Yes**	U	**Yes**	No	No	No	U	U	U	U	U	U	U	**Yes**	**Yes**	U	**Yes**	Corpuscallosum abnormalities
Hypotonia	Yes	No	No	Yes	U	No	Yes	Yes	Yes	No	Yes	No	No	No	U	U	U	U	U	U	U	Yes	No	No	No	
Seizures	Yes	No	No	No	U	No	No	No	No^b^	No	Yes	No	No	No	U	U	U	U	U	U	U	No	No	Yes	Yes	
Behavioural	No	No	Yes	No	U	U	Yes	U	U	U	Yes	U	U	Yes	U	U	U	U	U	U	U	U	Yes	U	Yes	
Other	No	Yes	No	No	U	Yes	Yes	No	Yes	No	No	No	No	Yes	U	U	U	U	U	U	U	Yes	No	U	No	
Congenital anomalies																										10/13
Facial features	Yes	Yes	Yes	Yes	U	U	No	Yes	Yes	Yes	Yes	No	Yes	No	U	U	U	U	U	No	Yes	U	U	U	U	Facial features
Cardiac	No	No	No	No	U	U	No	No	No	No	No	No	No	No	U	U	U	U	U	No	U	U	U	U	U	
Urogenital	No	No	No	No	U	U	Yes	No	No	No	No	No	No	No	U	U	U	U	U	No	U	U	U	U	U	
Gastrointestinal	No	No	No	No	U	U	No	No	No	No	No	No	No	No	U	U	U	U	U	No	U	U	U	U	U	
Other	No	No	Yes	Yes	U	U	Yes	No	Yes	No	No	No	Yes	Yes	U	U	U	U	U	U	U	U	U	U	U	

N, Normal; U, Unknown.

a Number of patients for which clinical characteristics are known.

b Febrile seizures. OFC ≤ 2 SD (e.g. microcephalic) and Corpus Callosum Abnormalities ‘Yes’ (e.g. present) highlighted in bold.

### 3.2 Homology modeling and analysis of *ZBTB18* missense variants

**Figure 3:**
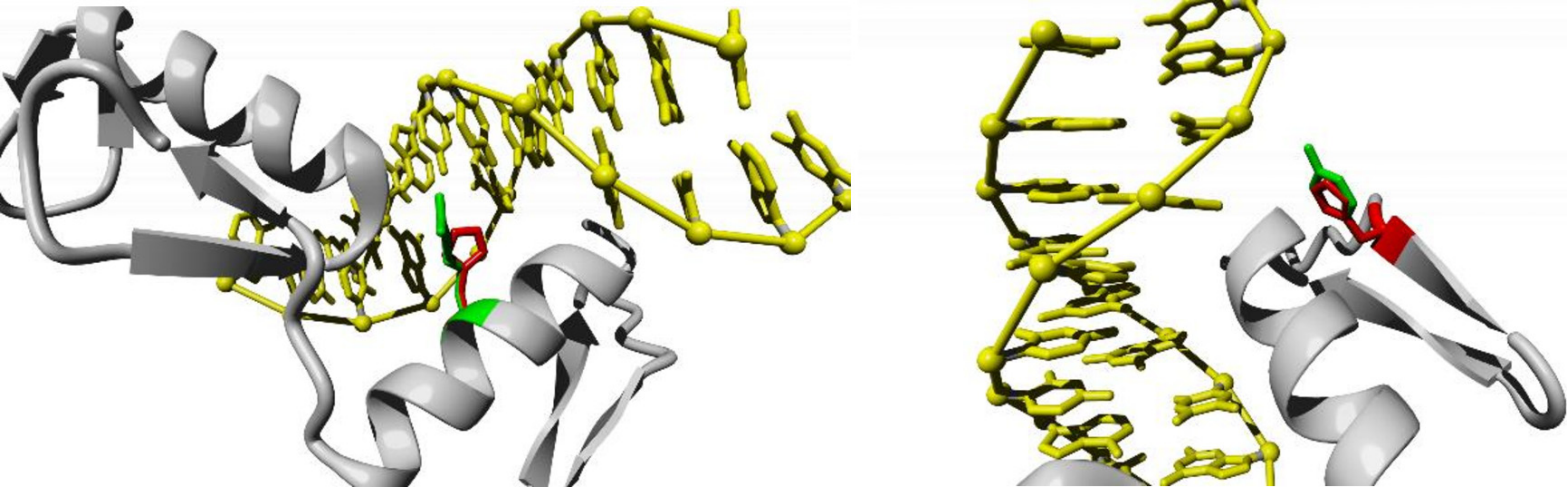
Close‐up of the recurrent de novo pathogenic variant Arg464His (left) and the pathogenic variant Tyr447His (right). The protein is colored gray, the side chains of both wild‐type and the mutant residue are shown and colored green and red, respectively

Variants in the ZNF domain of ZBTB18 were studied, using a homology model of this domain. The recurrent de novo p.(Arg464His) variant within the C2H2 ZNF domain of ZBTB18 may impair DNA‐binding properties of ZBTB18. In the wild‐type protein, the Arg464 residue is directed towards the major groove of the DNA and probably required for specific interactions between the transcription factor and the DNA. The substitution of this residue for a smaller and neutrally charged histidine might affect these interactions and thereby change the binding of the protein to its target DNA sequence (Figure 3 ‐ left).

The p.(Tyr447His) variant carried by patient 1 will affect tyrosine 447, which is pointed towards the phosphate backbone of the DNA. The possible hydrogen bonds between Y447 and the DNA will be lost because histidine is a smaller residue that will not be able to make the same interactions. This variant is therefore likely to affect the interaction between DNA and protein (Figure 3 ‐ right).

## 4 DISCUSSION

In this study, we confirm pathogenic variants in *ZBTB18* have major clinical consequences with an extensive and highly variable phenotypic impact. This is the most extensive review of clinical phenotypic features in patients with *ZBTB18* pathogenic variants to date and the first study to investigate the genotype‐phenotype correlation of LoF vs missense variants.

With ACC not being consistently present, we hypothesize that variants in *ZBTB18* are likely to cause a spectrum of structural corpus callosum abnormalities, that is, partial or global hypoplasia or agenesis in varying degrees. Other major features seen were developmental delay, dysmorphic facial features, and hypotonia.

Our data show no strong causal relation between *ZBTB18* pathogenic variants and seizures as this feature was present in only four out of 17 patients for whom seizure history information was available. This suggests that deletion of the *HNRNPU* gene is indeed an important contributor to this feature in patients with 1q43‐44 deletions (Hamdan et al., 2014; de Kovel et al., 2016; Depienne et al., 2017). Still, epilepsy is a feature of the *ZBTB18*‐related genetic disorder and a role for *ZBTB18* haploinsufficiency in epileptogenesis can therefore not be definitely excluded based on our data.

We explored a possible genotype‐phenotype correlation by the two putative mechanisms by which LoF (nonsense or frameshift) variants and missense variants cause alterations in the DNA‐binding capacity of ZBTB18. We did not find a clear genotype‐phenotype correlation with regard to position of the variant or type of the variant (i.e., LoF vs. missense).

ZBTB18 has a key role in cortical development, which probably explains the ID seen in patients carrying a pathogenic variant in this gene (Cohen et al., 2017). ZBTB18 functions as a transcriptional repressor influencing neuronal growth, differentiation, and maturation (Baubet et al., 2012; Ohtaka‐Maruyama et al., 2013; Okado et al., 2009). It represses *PAX6* (OMIM# 607108), *NEUROG2* (OMIM# 606624) and *NEUROD1* (OMIM# 601724). Expression of these three sequential proneurogenic genes causes intermediate neurogenic progenitors (INP) to differentiate and migrate. Importance of time‐ and place‐specific expression is hypothesized (Xiang et al., 2012). Loss of function of ZBTB18‐driven gene control could lead to early loss of transcriptional repression. Consequently, a premature differentiation and migration of INP's in this area, with subsequent disturbed neuronal development is to be expected.

ZBTB18 shows strong evolutionary conservation. For example, the overall human protein identity is 78% with *Danio rerio* and 99.4% with *M. musculus*, with even higher identity in the C2H2 ZNF domains. The protein is predicted to be highly intolerant to loss‐of‐function (LoF) variants, as is evident from the absence of LoF variants in ExAC and a high probability of loss‐of‐function Intolerance (pLI) of 0.97 (exac.broadinstitute.org; Lek et al. 2016) and a high‐rank haploinsufficiency score of 8.37 (Ni Huang, Lee, Marcotte, & Hurles, 2010).

Nonsense and frameshift variants lead to a premature termination codon in the last (second) exon of the *ZBTB18* gene (Kuzmiak & Maquat, 2006). Nonsense‐mediated decay (NMD) of *ZBTB18* mRNA is not expected to occur for these variants and they will probably lead to truncated proteins without the C2H2 ZNF domain. The eight nonsense and three out of five frameshift variants, we report are situated before or in Znf1. These pathogenic variants cause loss of DNA‐binding domains and the expected truncated proteins are likely to be dysfunctional. Subsequent haploinsufficiency is therefore most likely the cause of the observed phenotypic spectrum in patients with nonsense or frameshift variants in *ZBTB18*. We suggest this to be a more plausible explanation than a theoretical dominant negative effect or gain of function of the truncated protein, in particular given the high‐rank pLI and haploinsufficiency scores mentioned above.

By homology modeling, we illustrated a possible alteration in function caused by missense variants. *ZBTB18* encodes for a protein with four C2H2 Zinc fingers, structures found in nearly half of DNA‐binding factors and known to be involved in numerous biological processes (Persikov et al., 2015). Our homology model shows how the recurrent p.Arg464His substituting variant in the C2H2 structure of ZNF 3 probably affects DNA‐binding properties. Impaired binding of ZBTB18 to DNA will disturb its function as transcriptional repressor. This could lead to an increase in transcription of ZBTB18 target genes, similar to LoF‐based haploinsufficiency caused by truncating variants. Of the four patients identified thus far with the recurrent de novo p.Arg464His variant, two showed corpus callosum abnormalities while the other two did not undergo MRI imaging. A third patient (Cohen et al., 2017) with a structural anomaly of the corpus callosum carried a missense variant in the ZNF3 domain. The homology model of another (missense) variation (p.Tyr447His) in the zinc finger domains proved to cause a less distinctive change compared to the wildtype, coherent with a less pronounced phenotype.

We note that a very similar distribution of variants was recently documented for the *YY1* gene (OMIM# 600013), which also encodes for a transcription factor with four C2H2‐type zinc fingers. Strikingly, the missense variants in the *YY1* gene that cause intellectual disability, also appear to cluster in the zinc finger domains, with different variants affecting the identical amino acid residue (Gabriele et al., 2017). Mutational clustering in the ZNF region is described for *ZBTB20* (OMIM# 606025) as well, where de novo variants in the C2H2 ZNF domain of this protein lead to a hypothyroidism phenotype (Mattioli et al., 2016). In *ZBTB42* (OMIM# 613915), a p.Arg>His in one of its C2H2 ZNF domains causes a lethal congenital contracture syndrome (Patel et al., 2014). *Liu* et al. (2016) described functional characterization of variants in the ZNF region of *ZBTB7A* (OMIM# 605878) and invariably demonstrated loss of function. We expect clustering of missense variants in the C2H2 regions of other genes with homologous protein domains (Wiel, Venselaar, Veltman, Vriend, & Gilissen, 2017), causing phenotypic features by disabling proper DNA binding as well.

In conclusion, our data contribute to further delineate the heterogeneous phenotype of the *ZBTB18‐*related disorder. Homology modeling points to a variable degree of LoF caused by missense variants in C2H2 domains of *ZBTB18*. A lesser degree of LoF will present with a less distinctive phenotype. Complete LoF however, as can be seen in truncating variants, will present with an extensive variable phenotypic spectrum in which we could not define a clear genotype to phenotype presentation.

## CONFLICT OF INTEREST

None declared.
